# Improved Intelligence, Literacy and Mathematic Skills Following School-Based Intervention for Children in Foster Care

**DOI:** 10.3389/fpsyg.2020.00718

**Published:** 2020-04-24

**Authors:** Rikard Tordön, Marie Bladh, Gunilla Sydsjö, Carl Göran Svedin

**Affiliations:** ^1^Department of Biomedical and Clinical Sciences, Linköping University, Linköping, Sweden; ^2^Department of Social Sciences, Ersta Sköndal Bräcke University College, Stockholm, Sweden

**Keywords:** out-of-home care, intervention, assessment, intelligence, mathematics, literacy

## Abstract

Interventions aimed at improving school performance for children in foster care are few and are generally not implemented. By preventing failure in school, the prospect of reducing the risk for future poor health, substance abuse, unemployment, and other detrimental social conditions are met. This paper focuses on the change of preconditions for compulsory school performance in out-of-home care children, following an intervention called “Skolfam” that aims to improve school performance by individual assessments and school-based interventions. In this study, data were compiled from prospective repeated tests of 475 children in foster care in Sweden. Educational preconditions were analysed for compulsory school performance, such as intelligence (WISC-IV), psychosocial (SDQ) and adaptive behavior (ABAS-II), literacy (Reading Chains) and mathematical skills (Magne Mathematic Diagnoses) before and after the first 2 years of the “Skolfam” intervention. All tests were age-standardized and performed by experienced professionals. The results showed improved skills in complex aspects of literacy, mathematics, and cognitive performance, but no improvement in less complex literacy skills, adaptive behavior or mental health symptoms. In conclusion, higher-order cognitive functions can develop positively when appropriate school support is provided. Affective function, adaptive behavior, and psychosocial well-being present a more pervasive challenge for children in foster care. Implications for future research, practice in social services, and school is that further development of methods to aid future prospects for children in out-of-home care should aim to improve both cognitive higher-order executive-, and affective functions.

## Introduction

Children placed in out-of-home care are exposed to higher risk of poor outcomes with respect to health (Teyhan et al., 2018), physical and sexual abuse (Tordön et al., 2019), and substance abuse (Kobulsky, 2019). Also, these children have a poor outcome in terms of school performance (Berlin et al., 2011; Romano et al., 2015). Typically, incremental steps in school performance widen the gap with peers who are not in out-of-home care and differences become more pronounced when the children reach high school and college (Jackson and Cameron, 2012). These children are also subject to more actions when the school situation does not work as expected, for example being subject to more special education, retention and disciplinary actions (Scherr, 2007).

In a developed and modern society, high-level educational achievement can benefit both the individual as well as society (Dee, 2004). Also, an association between school achievements and a reduction of risk of adverse outcomes for children in out-of-home care has been found in quantitative register and qualitative method studies (Cameron et al., 2012; Forsman et al., 2016).

In the study by Berlin et al. (2011), the risks for suicide attempts, substance abuse, serious criminality, and public welfare dependency were reduced by 38–52% when controlling for poor school performance in the final compulsory school year. The authors concluded that “If society wants to improve life opportunities for care leavers, it is necessary to give them effective help with their schooling and education while they are in care” (Berlin et al., 2011, p. 2496). Interventions aimed at improving school performance for children in out-of-home care have been developed and, to a limited extent, evaluated. A review by Forsman and Vinnerljung (2012) found 11 studies on interventions, of which nine reported positive results. Liabo et al. (2013) found promising interventions but highlighted the lack of evidence for efficacy in interventions. In a more recent review, Männistö and Pirttimaa (2018), found 19 studies of interventions aimed at supporting school performance. They concluded that there was sufficient and robust evidence to support the practice and policy-making needed to strengthen foster care children’s educational and socio-emotional development. However, they also stated that there was still a need to improve both the quality and quantity of research to evaluate the interventions.

Interventions aimed at improving school performance for children in foster care vary in their design and setting. Some operate on a strategic level by appointing liaison persons (Zetlin et al., 2004) or resource coordination managers, such as the Virtual School Head Pilot^1^. Others try to enhance literacy and numeracy skills by using books or games as gifts that are mailed directly to the foster child, such as “Letterbox Club” (Griffiths, 2012). Some provide extra-curricular study support by providing tutoring assistance, by instructing foster parents on how to enhance their ability to support children in performing their school tasks (Vinnerljung et al., 2014; Hickey and Flynn, 2019). Some interventions enhance skills by engaging external resources such as voluntary university students, using a specific Direct Instruction method delivered in a group setting (Harper and Schmidt, 2012).

Children growing up in poor socioeconomic conditions (Franzén et al., 2008; Cameron et al., 2012; Simkiss et al., 2013), or children of parents with low educational attainment (Vinnerljung et al., 2005) are overrepresented in out-of-home care. The associations have been found to be stronger for younger children. Socioeconomic disadvantages have also been found to be robust over time. These disadvantages influence long-term trajectories of work- and health-related disadvantages for children investigated for social care interventions, or with earlier experiences of out-of-home care (Brännström et al., 2017; Almquist and Brännström, 2019).

Disruptions in family and change of school are other factors that affect children in foster care more than their peers. In a comprehensive study of factors that mediate school performance, Hattie (2008) found that a change of school influenced performance with a moderate effect size and was associated with developing new peer relations. In a study by Lewis et al. (2007) on adopted children with and without the experience of placement instability, the authors found associations between instability in early placements and adverse effects on social-emotional development.

The experience of previous success in school may also be an important factor. Hattie (2008) concluded that earlier acquired study skills have a great effect on scholastic performance. Also, the conclusions from the pan-European YiPPEE study “Young people in Public Care: Pathways to Education in Europe” (Jackson and Cameron, 2012), stressed the importance of early school success experiences as well as the detrimental effects of school changes.

There are other factors that could be regarded as pre-care adversities or disruptions that have a long-lasting influence on cognitive and intellectual abilities through school. In a study of the effects of early psychosocial deprivation on memory and executive function, Bos et al. (2009) found a detrimental impact on visual memory and executive functioning following early years of psychosocial deprivation in institutional care. When comparing 8-year-old children that were randomly assigned to foster care with those remaining in institutional care, no significant differences regarding measures of memory or executive functioning were found. This suggests that there might be traits of early adversities that are more resistant to change, where foster care alone does not provide an adequate remedy.

In a review of literature concerning the effects of early life stress on humans, Pechtel and Pizzagalli (2011, p. 55) concluded that “higher-order, complex cognitive and affective functions associated with brain regions undergoing protracted postnatal development are particularly vulnerable to the deleterious effects of early life stress.” Particularly affective deficits appeared to persist years after the stressor(-s) had ceased, while higher-order cognitive functions were to some extent, restored.

There is a need for well-conducted intervention studies aiming to improve school performance for children in out-of-home care, i.e., studies covering large populations with baseline measurements and with adequate follow-up times with the use of validated instruments.

Thus, the aim of the present study was to explore how literacy and mathematical skills, adaptive behavior, intelligence, and psychosocial strengths and difficulties change over the first 2 years of a school-based intervention aimed at improving school performance for children in foster care.

## Materials and Methods

### The Skolfam Working Model

In Sweden, a working model called Skolfam, (*School effort in Family care*), was developed in 2005. The objective was to improve compulsory school results for children in foster care by individual assessments, and cross-professional and cross-agency collaboration, followed by consultative support for school staff and foster parents and monitoring of the children’s progress. The first pilot was evaluated by Tideman et al. (2011), replicated by Tordön et al. (2014) and evaluated nationally in a quasi-experimental design by Durbeej and Hellner (2017). Durbeej and Hellner compared 54 foster care children in Skolfam to 37 in the comparison group, where the latter group did not receive any extra interventions other than regular school resources. Despite the small samples and a short timeframe for measuring improvements, the authors concluded: “the model may serve as a protective factor against adverse outcomes” (Durbeej and Hellner, 2017, p. 475).

Skolfam is staged in the ordinary school environment, using existing teachers and other school resources for interventions, but adding individual assessments with age-standardized instruments of foster children’s literacy, numeracy, intelligence, adaptive behavior, and psychosocial condition. It is a manualized model emphasizing collaboration between social services and schools by forming a team comprising a psychologist, a special education teacher, and the social service officers of both the child and the foster parents. After baseline assessment, a plan is made jointly by the school, the Skolfam team and the foster family, including the child. The objective is to fill knowledge gaps and optimize teaching based on objectively and individually assessed prerequisites. The team monitors progress by regular meetings in school and provides consultative support to staff and on school-related issues in the foster family when needed. A follow-up assessment, using the same age-standardized instruments, is performed after the first 2 years. In this follow-up, the progress is evaluated and, if necessary, the plan for the remainder of compulsory school is adjusted.

### Participants

**FIGURE 1:**
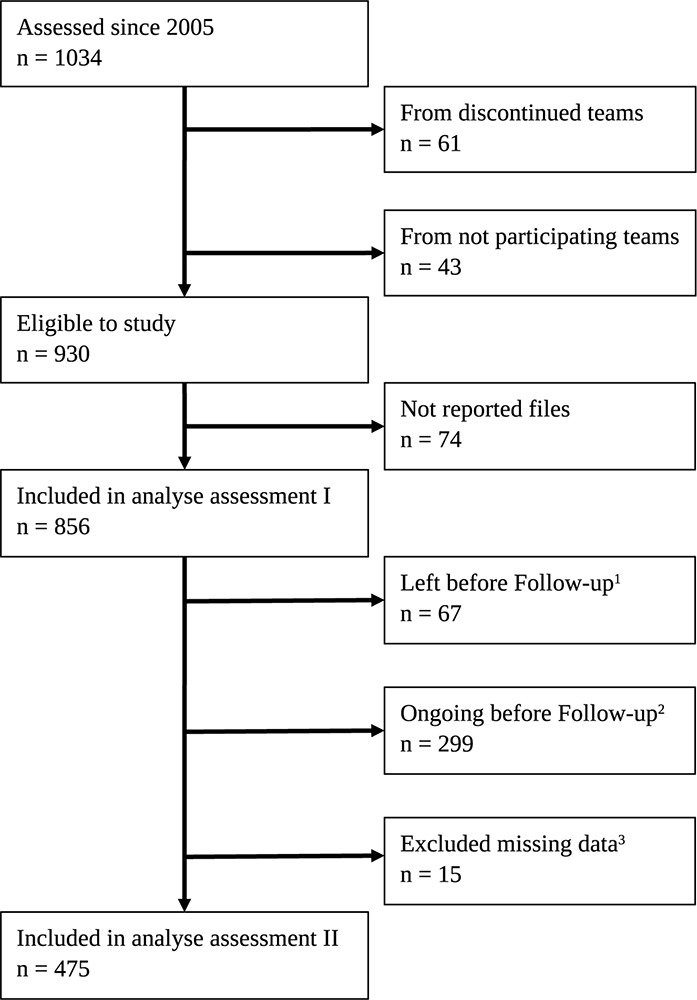
Flowchart of participants. (1) Reasons for leaving Skolfam can be a return to parent care, the foster parents becoming legal caregivers, or transfer to residential or institutional care. (2) Children assessed once but have not yet reached the time for 2-year follow-up. (3) Excluded cases due to extensive missing data, or missing test dates.

Since the start in 2005, to 2018, 1034 children had been included in Skolfam in 25 different Swedish municipalities with a total of 39 Skolfam teams, whereof 22 municipalities participated in the study. Data from 104 children were not available due to teams no longer being operative or not participating. Another 74 data sets were missing in the compilation from reporting teams. An analysis of the 856 cases with at least one baseline assessment was done and reported separately (Tordön et al., 2020). When out-of-home care ends, either by the care returning to a birth parent or by legally transferring the caregiver responsibility to foster parents, the Skolfam intervention is usually terminated. This was the case for 67 of the children in our sample. Of the children in Skolfam, 475 had in September 2018 been in the intervention long enough to be assessed twice, thereby providing data for pairwise tests of how prerequisites for school performance develop in the first 2 years with an individually adapted, school-based intervention, aimed at improving school performance (see Figure 1).

A dropout analysis of the cases that were included in the first assessment but dropped out of Skolfam before the second assessment (*n* = 67) showed no mean difference (*p* = 0.106–0.929) to the follow-up group in regard to the test results from the first assessments.

Inclusion criteria for children in the Skolfam intervention, and subsequently for the study were:

• Placed in foster care by a municipality committed to the *Skolfam* working model, regardless of residing in the same municipality or another.
• Placed in potential long-term foster care.
• In preschool-class (typically age six) to seventh year of compulsory school (typically age 13) at inclusion.

Exclusion criteria were:

• Placed in a short term-/acute-/temporary foster home.
• Meeting the criteria for inclusion in school for children with learning disabilities due to mental retardation.

There were no other selection criteria or prioritizations advised by the regulations in the manual.

Children remain in Skolfam until the end of compulsory school in the ninth grade, unless the intervention is terminated due to a change in the legal caregiver condition. In Sweden, the transfer of the legal caregiver to foster parents is the common policy, whereas other countries’ policies more commonly stipulate native adoption.

Gender distribution, school-year, native language, number of placements, and length of placement are presented in Table 1.

**TABLE 1:** Socio-demographic background data. *N* = 475.

	N	%
**Gender**		
Boy	250	52.6
Girl	225	47.4
**Native language**		
Swedish	327	68.8
Non-Swedish	117	24.6
Unknown	31	6.5
**School year at assessment II**		
2–3 (age 8–9)	83	17.5
4–6 (age 10–12)	202	42.6
7–9 (age 13–15)	142	29.9
10 or first-year upper secondary	2	0.4
Unknown	46	9.7
**No. placements**		
1	126	26.5
2	82	17.3
3 or more	80	16.8
Unknown	187	39.4
**Placement length (months), mean/SD**	69.08/34.63	
**Placement length (months), median/min-max**	60/25.5–172	

### Ethical Approval

In order to take part in Skolfam, legal caregivers, predominantly birth parents, provide written consent after receiving information about the working model, including a statement that depersonalized data will be collected for research purposes. For children cared for under compulsory law, the consent can be given by the appointed social officer *in loco parentis*. Children themselves are also given the right to decline participation in parts of, or the complete intervention. The Regional Ethical Board of Linköping approved the study on March 20th, 2018, registration number 2018/96-31.

### Measures

The Skolfam assessment battery aims primarily to aid the assessment of children’s prerequisites for school and to evaluate progress, and as a secondary aim, to aggregate research data. Aside from the tests and questionnaires included in the study, the full assessment is based on background interviews with foster parents, teachers, reviews of documentation in school healthcare and social service journals, and interviews with the child. In this study, data that could be operationalized and analyzed systematically were compiled and no additional qualitative data were collected. Assessments were performed for the first time when the child was included in Skolfam and were repeated after 24 months. Due to the naturalistic study design, and due to some children’s wish not to participate in parts of Skolfam, there was internal data dropout. This dropout varied between different tests and scales, thus it was not possible to perform full assessments with all instruments as stated by the Skolfam manual. Also, literacy tests typically have narrow age spans, measuring different aspects of literacy development of different ages, which also leads to high variance between different tests in the number of cases.

The validated and standardized instruments used in Skolfam are Swedish versions of:

**The Wechsler Intelligence Scale for Children (WISC), editions III and IV** (Wechsler, 1991, 2003). The WISC assesses intelligence using different composite index scales and as a full-scale intelligence gradient on a standardized score with a fixed mean of 100 and a fixed range of 15 points for one standard deviation (hereafter SD). Wechsler intelligence tests have been used in most countries since the 1950s (Wechsler, 1949) and are considered as reliable in measuring intelligence according to the theoretical model chosen by their designer (Kaufman et al., 2016; Canivez et al., 2017). Internal consistency coefficients for the WISC-IV indexes are 0.94 for the Verbal Function Index, 0.92 for the Perceptual Reasoning and Working Memory Indexes, 0.88 for Processing Speed Index, and 0.97 for the Full Scale Index (The Psychological Corporation, 2003, Table 4.1, p. 34). Validity, in terms of corrected correlation coefficients to previous version was (*r*_12_ = 0.89) in the Full Scale Index (Williams et al., 2003). All WISC assessments were performed by the Skolfam team’s psychologist.

The first 25 children from 2005 to 2008 were assessed using the third edition of WISC. From 2008 and onward, the fourth edition was used. Some index scales overlap between versions, and these data have been combined in the analyses. From 2017, the use of the fifth edition started in Skolfam municipalities, but none of these cases had gone through the follow-up assessments before data collection during autumn 2018 and were therefore not included in the current study.

**The Adaptive Behavior Assessment System (ABAS-II), version II. The ABAS-II** assesses adaptive behavior in nine domains, presented in conceptual, social and practical composite indexes and a general ability composite index, using the Wechsler scale. The assessments are performed by parents and teachers and compiled by the team’s psychologist. In Skolfam, foster parents answer the parent questionnaire. Adaptive behavior is sometimes referred to as *daily life skills*, reflecting how an individual can adapt behavior to cope with different conditions or tasks in life (Harrison and Oakland, 2008). Internal consistency in the ABAS-II is high, with reliability coefficients of 0.85–0.99 for the General Adaptive Composite, and the three adaptive domains (Oakland, 2011). Test-retest reliability is above 0.80. The construct validity is strong in factor analyses and it has shown a strong concurrent validity, *r* = 0.82, with the Vineland Adaptive Behavior Scales (Harrison and Oakland, 2008).

**The Strengths and Difficulties Questionnaire, Swedish (SDQ)**. Strengths and difficulties were assessed by the SDQ (Goodman, 1997) and compared to the United Kingdom teacher test norms (Meltzer et al., 2003), due to the lack of Swedish norms for teacher rating, and the most recent Swedish parent norms (Bjornsdotter et al., 2013). The instrument has 25 items in four scales reflecting problems (*emotional problems, conduct problems, hyperactivity*, and *peer relations*) and one strength scale (*prosocial behavior*). The range is 0–10 in each scale and 0–40 in the *total difficulties* score, with a higher score reflecting more problems, except for the prosocial behavior scale where scores are inverted and not included in the total score. The SDQ has showed a good reliability in the Swedish normative study of paper-and-pen versus Internet administered version by Bjornsdotter et al. (2013), with internal consistency in the subscales (polychoric ordinal alpha) ranging from 0.85 to 0.91. Teacher assessment reliability and validity was investigated by van den Heuvel et al. (2017). They report the internal consistency to alpha 0.80 for the total difficulties scale. Concurrent validity to the Teacher Report Form was found strong in all subscales, ranging from 0.54 to 0.73, with exception for the peer problems subscale, 0.46. The SDQ is compiled by the team psychologist.

**Reading Chains [Swedish “Läskedjor”] versions I and II.** Reading chains are primarily aimed at assessing skills in visual decoding of letters/digits, words or sentences. During the years 2005 to 2014 the first edition of these tests was used (Jacobson, 2001), and from 2014 Reading Chains-2 was used (Jacobson, 2014). *Letter chains* were used instead of *Digit chains* in the first school year and *Sentence chains* from the second school year, explaining the variance in the number of cases. There are no norm means or SDs spanning all school years reported from the standardization studies in the literacy tests, making a more precise calculation impossible. These tests are performed by the Skolfam team’s special education teacher. The tests in language and mathematics skills use the Stanine scale one – nine, with five as the mean and two scale steps representing one SD.

**Diagnosis in Reading and Spelling (Swedish “DLS”)**. DLS is a test for reading and writing skills, more broadly aimed than just visual decoding, with subtests developed by Järpsten and Taube (2002) including Word comprehension, Reading, Reading comprehension, Spelling, and Reading speed. The sub-test Reading is taken in school years one and two, Spelling and Word comprehension in school years three to nine, Reading comprehension in school years two and three, and Reading speed in school-years four to nine. DLS tests are performed by the Skolfam team’s special education teacher.

**Reading and Spelling (Swedish “LäSt”) for decoding and reading index.** The LäSt reading index is a test measuring text decoding of non-words and words, which reports a result in percentiles reflecting these aspects called the “Reading index.” This test was developed by Elwér et al. (2013). In the Skolfam setting, LäSt was used in school years one to five but occasionally also in the sixth year. LäSt tests are performed by the Skolfam team’s special education teacher.

**Olof Magne Mathematics Diagnoses (Swedish).** Numeracy skills were assessed with the Olof Magne mathematics series of tests. These tests were standardized in three studies 1977, 1986, and 2002 in a municipality with around 2,000 pupils in compulsory school (Engström and Magne, 2003). The Magne diagnostic tests aim to assess numeracy skills in different areas, such as number or quantity perception, number values, position, basic algebra, units and applied numeracy comprehension. Internal consistency in the normative study ranged from alpha 0.89 in the diagnoses for third school-year, to 0.97 in the diagnoses for seventh school-year (Engström and Magne, 2003). The Magne mathematics tests are performed by the Skolfam team’s special education teacher.

The Skolfam manual, in Swedish, is available online, at www.skolfam.se/artiklar-och-rapporter-om-skolfam/.

### Procedure

The procedure for testing foster care children in Skolfam is described in the manual. It is left to the team’s discretion to decide in cooperation with the child when and where the tests are conducted. Typically, the tests performed by the special education teacher are done in one session and those performed by the psychologists in another session. In some cases, these two sessions are done in one single day. If the child show signs of assessment fatigue, another session is scheduled to avoid bias from fatigue and assure optimal test conditions.

All municipalities in the Skolfam national network 2018 were sent a letter inviting them to participate. In addition to the invitation to participate, the letter also included instructions. Three municipalities could not participate due to temporary staff vacancies. In each municipality, the team or teams compiled data from their tests in an Excel template for each child along with the name of the team. They gave each child a unique code number and then transferred the data to the researcher who merged each individual file into a complete data file containing all anonymous data. For integrity reasons, background data was limited to test dates, gender, school-year at tests one and two, native language “Swedish” or “other,” number of placements and length of placement in months. No further actions engaging children were taken in order to compile data.

### Statistical Analyses

Comparable index scales in WISC-III and WISC-IV, *verbal comprehension* and *full scale* were combined. Categorical data are presented as numbers (n) and percent (%). Means for scales in the different instruments were calculated along with SD, and compared to norm groups from sources either in test manuals or from peer-reviewed published literature. The data is presented with the sample size (n) available for each sub-scale. Paired sample tests for means were performed to calculate differences. Where statistically significant (*p* < 0.05, two-sided) differences between first (T_1_) and second assessments (T_2_) were discovered, a calculation of effect size (d) was carried out, using a within-subjects calculator online, https://memory.psych.mun.ca/models/stats/effect_size.shtml.

The statistical analyses were performed using IBM SPSS Statistics 25 (IBM Inc., Armonk, NY, United States).

## Results

### Intelligence

Intelligence, as assessed with the WISC-IV full-scale index, showed at the first baseline assessment more than 0.5 SD under the mean for the age-standardized norm in the population. We also found an atypical profile variance of 12.66 points between a substantially lower working memory index mean and a perceptual reasoning mean close to the norm (see Table 2).

**TABLE 2:** Comparison of cognitive and adaptive behavior functioning between test 1 and test 2.

	Test I results n/mean/SD	Test II results mean/SD	Difference m2 – m1/SD	Paired samples test t/p	Effect size Cohens d^1^
**WISC-IV**					
Verbal comprehension	419/93.37/13.26	95.52/14.67	2.15/9.90	−4.45/<0.001	0.217
Perceptual reasoning	417/97.95/14.06	101.66/13.63	3.72/9.89	−7.67/<0.001	0.375
Working memory	418/85.29/13.14	88.74/13.59	3.45/12.00	−5.88/<0.001	0.287
Processing speed	418/92.58/13.12	94.94/13.78	2.36/11.19	−4.32/<0.001	0.211
Full scale IQ	414/91.14/12.79	94.73/13.40	3.59/8.35	−8.76/<0.001	0.423
**ABAS-II Teacher**					
Conceptual composite	320/85.61/19.61	84.40/19.91	−1.21/18.89	1.14/0.253	NA
Social composite	313/84.43/19.91	82.40/21.16	−2.03/21.20	1.70/0.091	NA
Practical composite	315/87.23/21.49	87.26/22.44	0.02/21.82	−0.18/0.986	NA
General ability comp.	331/85.60/20.26	85.26/21.58	−0.34/19.54	0.32/0.753	NA
**ABAS-II Foster parent**					
Conceptual composite	310/75.58/21.48	77.54/24.35	1.96/20.90	−1.65/0.100	NA
Social composite	310/75.99/19.98	76.92/21.39	0.93/18.83	−0.87/0.386	NA
Practical composite	310/83.69/19.13	84.35/21.17	0.66/20.63	−0.56/0.575	NA
General ability comp.	329/78.96/20.02	80.26/22.54	1.30/19.82	−1.19/0.234	NA

*^1^ Using Within-Subjects calculator at https://memory.psych.mun.ca/models/stats/effect_size.shtml.*

In all function indexes of WISC-IV, as well as the full-scale IQ index, the results showed improvements (*p* < 0.001) in the range of small to medium effect size after the first 2 years of the intervention. Results ranged from verbal comprehension index (*d* = 0.217) to perceptual reasoning index (*d* = 0.375). In the full-scale index, results improved with a medium effect size (*d* = 0.423) (see Table 2).

### Adaptive Behavior

Adaptive behavior also showed baseline means lower than the age-standardized norm, close to one full SD in teacher assessments in *general ability composite*. Foster parents’ assessment means were more than a full SD below norms in *general ability composite*. In comparison to the intelligence indexes, profile variance between sub-scale indexes was small in teachers’ assessments, with a 2.8 points difference between the lowest, *social composite* and the highest, *practical composite*. In foster parents’ assessments, the profile variance was higher with a difference of 8.11 points between the lowest, *conceptual composite* and the highest, *practical composite* (see Table 2).

Contrary to the results in intelligence, adaptive behavior did not change in any sub-scale or general ability index in either teachers’ or foster parents’ assessments. Means showed a tendency to decline in teachers’ assessments and there was a slight, but not significant incline in foster parents’ assessments (see Table 2).

### Language and Mathematics Skills

Language and mathematics skills at baseline were found to be approximately 0.5 SD lower than age-standardized means in *reading chains*, the *DLS test*, and the *Magne mathematics* diagnoses.

In *reading chains*, reflecting text decoding skills, letter-, digit-, and word chains showed no significant changes between T_1_ and T_2_. *Sentence chains*, however, increased (*p* = 0.001, *d* = 0.211) (see Table 3).

**TABLE 3:** Comparison of language and numeracy skills between test 1 and test 2.

	Test I results n/mean/SD	Test II results mean/SD	Difference m2 – m1/SD	Paired samples test t/p	Effect size Cohens d^1^
**Reading Chains**					
Letter Chains	70/3.99/1.49	4.29/1.65	0.30/1.43	1.76/0.083	NA
Digit Chains	178/3.88/1.93	4.07/1.91	0.19/1.76	1.45/0.149	NA
Word Chains	351/3.96/2.00	3.93/1.86	−0.03/1.63	−0.33/0.743	NA
Sentence Chains	234/3.71/1.78	4.01/1.73	0.30/1.42	3.26/0.001	0.211
**DLS (Read and Write)**					
Word comprehension	165/4.20/1.97	4.24/1.90	0.04/1.78	0.31/0.760	NA
Read comprehension	59/3.56/1.86	4.22/1.92	0.66/1.82	2.80/0.007	0.364
Spelling	341/4.25/2.06	4.45/2.07	0.20/1.57	2.38/0.018	0.127
Reading speed	212/4.13/1.98	3.92/2.01	−0.21/1.47	−2.10/0.037	0.143
**LäSt**					
Reading Index	70/52.39/27.32	53.91/29.32	1.52/20.23	0.63/0.531	NA
**Magne Mathematics**					
Total score	406/3.43/2.22	3.87/2.19	0.43/2.27	3.86/<0.001	0.194

*^1^Using Within-Subjects calculator at https://memory.psych.mun.ca/models/stats/effect_size.shtml.*

Results in the *DLS test*, reflecting a more comprehensive aspect of reading and writing skills, showed no change between T_1_ and T_2_ in the *word comprehension* sub-test. The *reading comprehension* test improved (*p* = 0.007, *d* = 0.364) and the *DLS spelling* test also showed improvements (*p* = 0.018, *d* = 0.127). *Reading speed* means declined (*p* = 0.037, *d* = 0.143) (see Table 3).

Mathematics skills showed a marked lower stanine scale means (in relation to age-standardized population norms at baseline) and an improvement (*p* < 0.001, *d* = 1.194) during the first 2 years of the intervention (see Table 3).

### Strengths and Difficulties

Psychosocial well-being was assessed by the *SDQ -Strengths and Difficulties Questionnaire* for teachers’ and (foster-) parents’ assessments. At baseline, all sub-scales and the *total problems* scale showed higher levels (*p* < 0.001) compared to norms, and the *prosocial behavior* scale lower levels (*p* < 0.001) than norms, in teachers’ as well as in foster parents’ assessments. Norm values are included for comparison in Table 4.

**TABLE 4:** Comparisons of strengths and difficulties between test 1 and test 2.

	Norm data n/mean/SD	Test I results n/mean/SD	Test II results mean/SD	Difference m2 – m1/SD	Paired samples test t/p	Effect size Cohens d^1^
**SDQ Teacher^2^**						
Emotional symptoms	8208/1.4/1.9	337/1.91/2.08	1.94/2.08	0.02/2.20	0.210/0.834	NA
Conduct problems	8208/0.9/1.6	338/2.04/2.35	2.00/2.39	−0.05/2.34	−0.360/0.719	NA
Hyperactivity	8208/2.9/2.8	338/4.85/3.32	4.52/3.14	−0.33/2.85	−2.149/0.032	0.116
Peer problems	8208/1.4/1.8	338/2.19/2.28	2.23/2.36	0.04/2.46	0.287/0.774	NA
Prosocial behavior	8208/7.2/2.4	336/6.47/2.92	6.68/2.79	0.21/3.17	1.203/0.230	NA
SDQ total problems	8208/6.6/6.0	361/11.01/7.58	10.49/7.32	−0.52/6.93	−1.432/0.153	NA
**SDQ Foster parent^3^**						
Emotional symptoms	946/1.4/1.7	329/2.54/2.46	2.21/2.15	−0.33/2.24	2.672/0.008	0.148
Conduct problems	946/1.1/1.3	330/2.50/2.16	2.41/2.25	−0.08/2.03	0.745/0.457	NA
Hyperactivity	946/2.3/2.1	330/4.99/2.92	4.60/2.91	−0.39/2.51	2.822/0.005	0.156
Peer problems	946/1.2/1.5	330/2.57/2.48	2.52/2.40	−0.05/2.17	0.419/0.676	NA
Prosocial behavior	946/8.5/1.6	330/6.70/2.62	6.93/2.62	0.23/2.63	−1.583/0.114	NA
SDQ total problems	946/6.1/4.8	351/12.67/7.36	11.72/7.36	−0.95/6.10	2.912/0.004	0.156

*^1^ Using Within-Subjects calculator at https://memory.psych.mun.ca/models/stats/effect_size.shtml.^2^ Norm data from SDQ British standardization study, n = 8208 (Meltzer et al., 2003). ^3^ Norm data from Swedish standardization study, n = 946 (Bjornsdotter et al., 2013).*

The results show a small decline (*p* = 0.032, *d* = 0.116) in the *hyperactivity* sub-scale in teachers’ assessments following the first 2 years of the intervention, but there was no significant change in any other sub-scale, the *total problems* scale, or in the *prosocial behavior* scale.

In the foster parents’ assessments, means declined (*p* = 0.008, *d* = 0.148) in the sub-scale *emotional symptoms*, and in the *hyperactivity* sub-scale (*p* = 0.005, *d* = 0.156). In the *total problems* scale, means declined (*p* = 0.004, *d* = 0.156).

Despite the decline in some of the SDQ sub-scales, means remained high, well above norms for the normal population in all sub- and total problems scales, and lower in the *prosocial* scale.

## Discussion

This study investigated how literacy and mathematical skills, adaptive behavior, intelligence, and psychosocial strengths and difficulties changed during 2 years of a school-based intervention aimed at improving school performance for children in foster care. The results can be summarized in four main findings.

### Intelligence and Adaptive Behavior

First, all indexes in the Wechsler scales showed improved results, with the largest effect size on the *perceptual reasoning* index and the smallest on the *processing speed* index. This is probably one of the first studies to report a positive development of the measured intelligence of children in foster care. Since there is no reference group of foster care children to compare with in our study, results are related to the study by Durbeej and Hellner (2017). In their study, the control group of children in foster care showed a tendency to decreased results in intelligence, literacy and mathematic skills, while the Skolfam group showed tendencies to improved scores over time. Another study of 2 453 children in the United States National Survey of Child and Adolescent Well-Being (Berger et al., 2009) found foster care alone having no influence on intelligence.

Providing individually adapted school-based training for under-stimulated children in foster care might facilitate their development of higher-order executive functions. To our knowledge, this positive effect has not previously been observed in the normal population or foster care children without specific academic interventions.

Second, contrary to expectations, changes in adaptive behavior were not apparent. The results remained on a level one full SD below the norm in the teachers’ assessments and even lower in the foster parents’ assessments. This finding contrasts with a study by Horwitz et al. (2001) in which a positive improvement was found over time in foster care.

The rationale underpinning the Skolfam design was that once deficits in overt behavior skills were revealed in the first assessment, it would be a reasonably easy task to train children specifically in those areas. This was meant to be achieved by using well-proven methods from behavioral psychology with instruction and reinforcement of desired behavior. But the results reveal difficulties in improving adaptive behavior among children in foster care in this sample.

There is a plausible reason for the absence of development in adaptive behavior since it is regarded as more sensitive to affective functions than to higher-order cognitive functions. As concluded by Pechtel and Pizzagalli (2011), both cognitive and affective functioning can be impaired by early life stress experiences. But when there seems to be a catch-up effect for high-order cognitive functions upon relief of the stressors, affective functioning appears to be more resistant to change. Attention deficits (McLaughlin et al., 2015) and inhibitory control, as suggested by Pears et al. (2015) can also be regarded as factors that could impact the ability to re-learn adaptive behaviors, providing another piece to understanding the lack of improvements in this domain.

### Literacy and Mathematic Skills

Third, the starting point for literacy and mathematics skills for children in our study was an average level in line with intelligence, around 0.5 SD below the normal population as expressed in the norms. There is growing support in the literature for a reciprocal relation between intelligence and mathematics (Cowan et al., 2018). The results from this study provide support for that hypothesis, meaning that development in mathematical ability can possibly influence intelligence, and developments in general cognitive ability can influence mathematics. How this process works has not yet been described. Other studies suggest reading skills contribute to the growth of general cognitive skills and vice versa (Ferrer et al., 2007; Ritchie et al., 2015). For our study the results in mathematics were limited to one school-year standardized stanine value of generic/general mathematic skills per test, whereas the wider range of literacy measures provided a more diversified analysis of different components.

One interesting point is that in literacy, no change in measurements of less complex skills such as letter-, digit- or word chains, or word comprehension was found. However, there was a positive development in more complex literacy skills such as sentence chains, reading comprehension and spelling. The latter set of more complex literacy skills requires more than mere decoding.

In the literacy tests, only reading speed showed decreased skills over the 2-year timeframe, suggesting no functional relation with processing speed in the cognitive tests, which increased. A plausible, but not proven explanation would be that children in Skolfam develop a quality reading strategy, deliberately slowing down their pace in order to comprehend text, rather than process as many words as possible.

### Psychosocial Well-Being

Fourth, the development of psychosocial well-being and function, based on assessments from teachers and foster parents using the SDQ, were more discouraging. The starting point at the first assessments revealed higher levels in all sub-scales reflecting problems, and a lower level in the prosocial behavior scale for children in foster home care. The results after the second assessment revealed significant improvements with lowered means in the teachers’ assessments in the sub-scale *hyperactivity*. This result makes sense in light of the positive development in literacy and numeracy skills, and the positive intellectual progress in the WISC assessments. Children who develop cognitive abilities and start to master the scholastic challenges would logically be perceived as less hyperactive.

In the foster parents’ assessments, *hyperactivity* along with means in *emotional symptoms* was found to decline, which in turn also led to a decrease in the *total problems scale*, since there were no increased means in other scales.

Nevertheless, means in teachers’ assessments are still high above those of peers in the scales *emotional symptoms*, *conduct-* and *peer problems*, and below peers in *prosocial behaviour*. And in foster parents’ assessments, the scores after intervention were still reflecting more problems in all sub-scales, close to the cut-off levels for clinical importance suggested by Goodman (1997).

Results from the analysis of the SDQ scores indicates that foster care, even with added interventions to aid good school progress, does not ensure broad psychosocial well-being for children in out-of-home placements, but it can contribute to reducing hyperactivity both in school and at home as well as emotional problems at home.

The mean levels and size of change between tests were similar to those found by Durbeej and Hellner (2017) in their quasi-experimental effect evaluation on a smaller portion in the same sample as ours, providing support for their conclusions. In their study, a matched comparison group of foster care children showed a negative development between tests, with increased means in the total problems scale and decreased means in the prosocial scale.

### Implications for Developing Interventions to Support Foster Children’s School Achievements

In general, most interventions aimed at strengthening school achievements for children in foster care show promising results, but they are too diverse in methodology or output measures to make systematic meta-analyses feasible, or they lack the statistical power needed to draw valid conclusions. Research on interventions in foster care is also an area with obstacles to staging high standard RCT studies for evaluation purposes, due to weak research infrastructure for controlled trials and culture in social sciences (Mezey et al., 2015) combined with the need for long timeframes for measurable effects.

One hypothesis to explain the diversity in the design rationale for interventions is that there is no scientific consensus on the most important causes of out-of-home care children’s educational underachievement in school. Depending on the idea of what causes underachievement, the understanding of the factors mediating school performance for children placed in foster care will vary, and subsequently so will the design of interventions. Our results reflect what can and cannot be achieved through school-based intervention for foster children in primary school. Skolfam has some core characteristics if comparisons of different methods are to be made in this respect. First, it has broad aims, targeting both literacy and mathematic skills in school, and pays attention to the whole environment although focusing on the child’s ability to perform at school. Second, it is ongoing over a long time, monitoring the progress through scheduled meetings in school until the end of primary school. Third, it involves all children in foster care in a population such as a municipality, regardless of whether there are any manifest problems or not. Skolfam has a selective group preventive design, not waiting to target problems with remedies when already visible.

### Limitations

The study utilizes a design without a control group for comparisons, which makes valid generalizations of effects not feasible. Instead, it reports on how conditions for school performance change in Swedish foster children who are provided with an intervention designed to address their prerequisites for school performance by more precise directed support in the school environment, following individual assessments. Another aspect that may impact the study validity is whether it has a retrospective or prospective design. All the tests that generated data for the study were performed prospectively, as the children entered the Skolfam intervention. The collection, compilation, and analysis were carried out retrospectively. This creates a risk for bias by skewed external dropout, but no signs, or remarks of any such skewness or attempts at selection in data were found or reported from the teams. The ability for children to decline participation in selective tests could skew the results through internal dropout. If so, the tests would probably be ones the child had previous poor experiences in. Thus, there would be fewer poor results, leading to higher means in our compilation and an underestimation of problems.

Another limitation is related to background data. The collected data have no information about the reasons for care, birth parents’ educational level, economic conditions or health status. There was also a fairly large internal data dropout regarding the number of placements (*N* = 187) and months in the present foster home. This was due to less rigorous routines for documenting information that did not have direct relevance for the operational assessments in the teams.

The study unfortunately lacks data regarding specific interventions given to children in Skolfam. Interventions were heterogeneous and varied from one school to another and over time. A coarse systematic frame to report widely defined interventions such as “intense reading training”, “special education support” or “working memory training” was included, but few teams had systematically noted provided interventions in their records.

The fact that data was collected several years after the intervention program has probably resulted in a larger external dropout than what would have been if collection of data had been done during intervention. On the other hand, this is not considered to have skewed results in a particular direction.

### Relevance and Implications

This study has several strengths related to relevance for practice. First, the naturalistic design, using prospectively collected data, provides a reliable base and well reflects the population of foster care children in compulsory school. Second, the large sample adds to the reliability of the analyses through ensuring good statistical power. Third, the use of widely used, age-standardized comprehensive instruments adds to methodological transparency and reproducibility, even though literacy and mathematics instruments would need to be nationally adapted.

Based on our findings, school interventions for children in foster care should focus on school matters such as mathematics, literacy and other factors related to a good study environment. Health-related aspects, affective functioning, attachment, and relational problems are of course important to address, but are not necessarily the only prerequisites for school performance, and they should not to be expected to improve via school interventions. Adding interventions aimed at addressing affective functioning and psychosocial well-being for children in foster care is recommended in the future based on the results of this study.

## Conclusion

This was a study of 475 foster care children in compulsory school, given a school-based preventive intervention aimed at strengthening school performance. Conclusions are that higher-order cognitive functions, such as mathematics skills, intelligence, and some aspects of literacy can develop in a positive direction when appropriate school support is provided constantly during compulsory school. Also, affective function, adaptive behavior, and psychosocial well-being present a more pervasive challenge that does not necessarily change when school performance does, nor can affective function or psychosocial well-being serve as sole prerequisites for school achievement, but they could possibly contribute. Thus, studies of the role of affective versus higher-order executive functions is an important field for the future development of interventions for children and youth in out-of-home care.

In short, Skolfam at least prevents further detrimental development in school, as stated by Durbeej and Hellner (2017), showing a small to medium effect after the first 2 years on intelligence, mathematics and higher-order executive aspects of literacy. Skolfam shows no measurable effect on variables sensitive to affective function, such as psychosocial well-being and adaptive behavior.

## Data Availability Statement

The datasets generated for this study are available on request to the corresponding author.

## Ethics Statement

The studies involving human participants were reviewed and approved by The Regional Ethical Board of Linköping March 20th 2018, reg. nr. 2018/96-31. Written informed consent to participate in this study was provided by the participants’ legal guardian/next of kin.

## Author Contributions

RT contributed to the design of the study, communicated internally with participating municipalities, applied for grants from the Children’s Welfare Foundation, compiled the data, contributed to statistical analyses, and wrote the manuscript. MB contributed to statistical analyses and to setting out the results in the tables. CS and GS supervised the design and ethical approval. All the authors have been involved in the manuscript writing and interpretation of the results.

## Conflict of Interest

The authors declare that the research was conducted in the absence of any commercial or financial relationships that could be construed as a potential conflict of interest.

## References

[B1] Almquist Y. B. Brännström L. (2019). Do trajectories of economic, work- and health-related disadvantages explain child welfare clients’ increased mortality risk? A prospective cohort study. *BMC Public Health* 19:418. 10.1186/s12889-019-6752-y 30999882 PMC6472010

[B2] Berger L. M. Bruch S. K. Johnson E. I. James S. Rubin D. (2009). Estimating the “impact” of out-of-home placement on child well-being: approaching the problem of selection bias. *Child Dev.* 80 1856–1876. 10.1111/j.1467-8624.2009.01372.x 19930356 PMC2836492

[B3] Berlin M. Vinnerljung B. Hjern A. (2011). School performance in primary school and psychosocial problems in young adulthood among care leavers from long term foster care. *Child. Youth Serv. Rev.* 33 2489–2497. 10.1016/j.childyouth.2011.08.024

[B4] Bjornsdotter A. Enebrink P. Ghaderi A. (2013). Psychometric properties of online administered parental strengths and difficulties questionnaire (SDQ), and normative data based on combined online and paper-and-pencil administration. *Child Adolescent Psychiatry Ment. Health* 7:40. 10.1186/1753-2000-7-40 24325882 PMC3898053

[B5] Bos K. J. Fox N. Zeanah C. H. Nelson C. A. III (2009). Effects of early psychosocial deprivation on the development of memory and executive function. *Front. Behav. Neurosc.* 3:16. 10.3389/neuro.08.016.2009 19750200 PMC2741295

[B6] Brännström L. Forsman H. Vinnerljung B. Almquist Y. B. (2017). The truly disadvantaged? Midlife outcome dynamics of individuals with experiences of out-of-home care. *Child Abuse Neglect* 67 408–418. 10.1016/j.chiabu.2016.11.009 27884505

[B7] Cameron C. Jackson S. Hauari H. Hollingworth K. (2012). Continuing educational participation among children in care in five countries: some issues of social class. *J. Educ. Policy* 27 387–399. 10.1080/02680939.2011.644811

[B8] Canivez G. L. Watkins M. W. Dombrowski S. C. (2017). Structural validity of the Wechsler Intelligence Scale for Children-Fifth Edition: confirmatory factor analyses with the 16 primary and secondary subtests. *Psychol. Assess.* 29 458–472. 10.1037/pas0000358 27442624

[B9] Cowan R. Hurry J. Midouhas E. (2018). The relationship between learning mathematics and general cognitive ability in primary school. *Br. J. Dev. Psychol.* 36 277–284. 10.1111/bjdp.12200 28801949

[B10] Dee T. S. (2004). Are there civic returns to education? *J. Public Econ.* 88 1697–1720. 10.1016/j.jpubeco.2003.11.002

[B11] Durbeej N. Hellner C. (2017). Improving school performance among Swedish foster children: a quasi experimental study exploring outcomes of the Skolfam model. *Child. Youth Serv. Rev.* 82 466–476. 10.1016/j.childyouth.2017.10.014

[B12] Elwér Å Fridolfsson I. Samuelsson S. Wiklund C. (2013). *LäSt - Test i läsförståelse, läsning och stavning.* Stockholm: Hogrefe Psykologiförlaget AB.

[B13] Engström A. Magne O. (2003). *Medelsta-Matematik Hur väl behärskar grundskolans elever lärostoffet enligt Lgr 69, Lgr 80 och Lpo 94?.* Örebro: Pedagogiska institutionen.

[B14] Ferrer E. McArdle J. J. Shaywitz B. A. Holahan J. M. Marchione K. Shaywitz S. E. (2007). Longitudinal models of developmental dynamics between reading and cognition from childhood to adolescence. *Dev. Psychol.* 43 1460–1473. 10.1037/0012-1649.43.6.1460 18020824

[B15] Forsman H. Brännström L. Vinnerljung B. Hjern A. (2016). Does poor school performance cause later psychosocial problems among children in foster care? Evidence from national longitudinal registry data. *Child Abuse Negl.* 57 61–71. 10.1016/j.chiabu.2016.06.006 27318971

[B16] Forsman H. Vinnerljung B. (2012). Interventions aiming to improve school achievements of children in out-of-home care: a scoping review. *Child. Youth Serv. Rev.* 34 1084–1091. 10.1016/j.childyouth.2012.01.037

[B17] Franzén E. Vinnerljung B. Hjern A. (2008). The epidemiology of out-of-home care for children and youth: a national cohort study. *Br. J. Soc. Work* 38 1043–1059. 10.1093/bjsw/bcl380

[B18] Goodman R. (1997). The strengths and difficulties questionnaire: a research note. *J. Child Psychol. Psychiatry* 38 581–586. 10.1111/j.1469-7610.1997.tb01545.x 9255702

[B19] Griffiths R. (2012). The Letterbox Club: an account of a postal club to raise the achievement of children aged 7 to 13 in foster care. *Child. Youth Serv. Rev.* 34 1101–1106. 10.1016/j.childyouth.2012.01.039

[B20] Harper J. Schmidt F. (2012). Preliminary effects of a group-based tutoring program for children in long-term foster care. *Child. Youth Serv. Rev.* 34 1176–1182. 10.1016/j.childyouth.2012.01.040

[B21] Harrison P. Oakland T. (2008). *Adaptive Behavior Assessment System - Second Edition [Swedish translation].* San Antonio, TX: Harcourt Assessment, Inc.

[B22] Hattie J. A. C. (2008). *Visible Learning: A Synthesis of Over 800 Meta-Analyses Relating to Achievement.* Abingdon: Routledge.

[B23] Hickey A. J. Flynn R. J. (2019). Effects of the TutorBright tutoring programme on the reading and mathematics skills of children in foster care: a randomised controlled trial. *Oxford Rev. Educ.* 45 519–537. 10.1080/03054985.2019.1607724

[B24] Horwitz A. V. Widom C. S. McLaughlin J. White H. R. (2001). The impact of childhood abuse and neglect on adult mental health: a prospective study. *J. Health Soc. Behav.* 42 184–201. 11467252

[B25] Jackson S. Cameron C. (2012). Leaving care: looking ahead and aiming higher. *Child. Youth Serv. Rev.* 34 1107–1114. 10.1016/j.childyouth.2012.01.041

[B26] Jacobson C. (2001). *Läskedjor Manual.* Stockholm: Hogrefe Psykologiförlaget AB.

[B27] Jacobson C. (2014). *LäsKedjor-2. För skolår 1 – år 1 i gymnasiet.* Stockholm: Hogrefe Psykologiförlaget AB.

[B28] Järpsten B. Taube K. (2002). *DLS Handledning för klasserna 4-6; DLS handledning för klasserna 2-3; DLS handledning för klasserna 4-6; DLS handledning för skolår 7-9 och år 1 i gymnasiet.* Stockholm: Hogrefe Psykologiförlaget AB.

[B29] Kaufman A. S. Flanagan D. P. Alfonso V. C. Mascolo J. T. (2016). Test review: wechsler intelligence scale for children, Fourth Edition (WISC-IV). *J. Psychoeduc. Assess.* 24 278–295. 10.1177/0734282906288389

[B30] Kobulsky J. M. (2019). The prevalence of substance use in child welfare and general population eighth graders in the United States. *Subst. Misuse* 54 1618–1626. 10.1080/10826084.2019.1594907 31008676

[B31] Lewis E. E. Dozier M. Ackerman J. Sepulveda-Kozakowski S. (2007). The effect of placement instability on adopted children’s inhibitory control abilities and oppositional behavior. *Dev. Psychol.* 43 1415–1427. 10.1037/0012-1649.43.6.1415 18020821

[B32] Liabo K. Gray K. Mulcahy D. (2013). A systematic review of interventions to support looked-after children in school. *Child Fam. Soc. Work* 18 341–353. 10.1111/j.1365-2206.2012.00850.x

[B33] Männistö I. I. Pirttimaa R. A. (2018). A review of interventions to support the educational attainments of children and adolescents in foster care. *Adopt. Foster.* 42 266–281. 10.1177/0308575918791627

[B34] McLaughlin K. A. Sheridan M. A. Tibu F. Fox N. A. Zeanah C. H. Nelson C. A. (2015). Causal effects of the early caregiving environment on development of stress response systems in children. *Proc. Natl. Acad. Sci. U.S.A.* 112 5637–5642. 10.1073/pnas.1423363112 25902515 PMC4426436

[B35] Meltzer H. Gatward R. Goodman R. Ford T. (2003). Mental health of children and adolescents in Great Britain. *Int. Rev. Psychiatry* 15 185–187. 10.1080/0954026021000046155 12745331

[B36] Mezey G. Robinson F. Campbell R. Gillard S. Macdonald G. Meyer D. (2015). Challenges to undertaking randomised trials with looked after children in social care settings. *Trials* 16:206. 10.1186/s13063-015-0708-z 25947202 PMC4486703

[B37] Oakland T. (2011). “Adaptive behavior assessment system – Second Edition,” in *Encyclopedia of Clinical Neuropsychology*, eds Kreutzer J. S. DeLuca J. Caplan B., (New York, NY: Springer New York), 37–39. 10.1007/978-0-387-79948-3_1506

[B38] Pears K. C. Kim H. K. Buchanan R. Fisher P. A. (2015). Adverse consequences of school mobility for children in foster care: a prospective longitudinal study. *Child Dev.* 86 1210–1226. 10.1111/cdev.12374 25906815 PMC4618793

[B39] Pechtel P. Pizzagalli D. A. (2011). Effects of early life stress on cognitive and affective function: an integrated review of human literature. *Psychopharmacology* 214 55–70. 10.1007/s00213-010-2009-2002 20865251 PMC3050094

[B40] Ritchie S. J. Bates T. C. Plomin R. (2015). Does learning to read improve intelligence? A longitudinal multivariate analysis in identical twins from age 7 to 16. *Child. Dev.* 86 23–36. 10.1111/cdev.12272 25056688 PMC4354297

[B41] Romano E. Babchishin L. Marquis R. Fréchette S. (2015). Childhood maltreatment and educational outcomes. *Trauma Violence Abuse* 16 418–437. 10.1177/1524838014537908 24920354

[B42] Scherr T. G. (2007). Educational experiences of children in foster care:meta-analyses of special education, retention and discipline rates. *Sch. Psychol. Int.* 28 419–436. 10.1177/0143034307084133

[B43] Simkiss D. E. Stallard N. Thorogood M. (2013). A systematic literature review of the risk factors associated with children entering public care. *Child Care Health Dev.* 39 628–642. 10.1111/cch.12010 23210455

[B44] Teyhan A. Wijedasa D. MacLeod J. (2018). Adult psychosocial outcomes of men and women who were looked-after or adopted as children: prospective observational study. *BMJ Open* 8:e019095. 10.1136/bmjopen-2017-019095 29439075 PMC5829744

[B45] The Psychological Corporation, (2003). *WISC-IV Technical and Interpretive Manual.* San Antonio, TX: The Psychological Corporation.

[B46] Tideman E. Vinnerljung B. Hintze K. Aldenius Isaksson A. (2011). Improving foster children’s school achievements: promising results from a swedish intensive study. *Adoption Fost.* 35 44–56. 10.1177/030857591103500106

[B47] Tordön R. Bladh M. Svedin C. G. Sydsjö G. (2020). Challenging intellectual, behavioral and educational prerequisites for interventions aimed at school aged children in foster care. A compilation of Swedish test results. *Child. Youth Serv. Rev.* 108:104598 10.1016/j.childyouth.2019.104598

[B48] Tordön R. Svedin C. G. Fredlund C. Jonsson L. Priebe G. Sydsjö G. (2019). Background, experience of abuse, and mental health among adolescents in out-of-home care: a cross-sectional study of a Swedish high school national sample. *Nord. J. Psychiatry* 73 16–23. 10.1080/08039488.2018.1527397 30561234

[B49] Tordön R. Vinnerljung B. Axelsson U. (2014). Improving foster children’s school performance: a replication of the Helsingborg study. *Adopt. Fost.* 38 37–48. 10.1177/0308575913518003

[B50] van den Heuvel M. Jansen D. E. M. C. Stewart R. E. Smits-Engelsman B. C. M. Reijneveld S. A. Flapper B. C. T. (2017). How reliable and valid is the teacher version of the Strengths and Difficulties Questionnaire in primary school children? *PLoS One* 12:e0176605. 10.1371/journal.pone.0176605 28453573 PMC5409073

[B51] Vinnerljung B. Öman M. Gunnarson T. (2005). Educational attainments of former child welfare clients - A SWEDISH national cohort study. *Int. J. Soc. Welfare* 14 265–276. 10.1111/j.1369-6866.2005.00369.x

[B52] Vinnerljung B. Tideman E. Sallnäs M. Forsman H. (2014). Paired Reading for foster children: results from a Swedish replication of an English literacy intervention. *Adopt. Fost.* 38 361–373. 10.1177/0308575914553543

[B53] Wechsler D. (1949). *Wechsler Intelligence Scale for Children.* San Antonio, TX: Psychological Corporation.

[B54] Wechsler D. (1991). *Manual for the Wechsler Intelligence Scale for Children-Third Edition.* San Antonio, TX: Psychological Corporation.

[B55] Wechsler D. (2003). *Wechsler Intelligence Scale for Children-Fourth Edition.* San Antonia, TX: PsychCorp.

[B56] Williams P. E. Weiss L. G. Rolfhus E. L. (2003). *WISC-IV Technical Report #2 Psychometric Properties.* San Antonia, TX: Pearson.

[B57] Zetlin A. Weinberg L. Kimm C. (2004). Improving education outcomes for children in foster care: intervention by an education liaison. *J. Educ. Stud. Placed Risk* 9 421–429. 10.1207/s15327671espr0904_5

